# Genetic and functional analyses implicate microRNA 499A in bipolar disorder development

**DOI:** 10.1038/s41398-022-02176-6

**Published:** 2022-10-07

**Authors:** Aileen Tielke, Helena Martins, Michael A. Pelzl, Anna Maaser-Hecker, Friederike S. David, Céline S. Reinbold, Fabian Streit, Lea Sirignano, Markus Schwarz, Helmut Vedder, Jutta Kammerer-Ciernioch, Margot Albus, Margitta Borrmann-Hassenbach, Martin Hautzinger, Karola Hünten, Franziska Degenhardt, Sascha B. Fischer, Eva C. Beins, Stefan Herms, Per Hoffmann, Thomas G. Schulze, Stephanie H. Witt, Marcella Rietschel, Sven Cichon, Markus M. Nöthen, Gerhard Schratt, Andreas J. Forstner

**Affiliations:** 1 Institute of Human Genetics, University of Bonn, School of Medicine & University Hospital Bonn, Bonn, Germany; 2 Salus Clinic Hürth, Hürth, Germany; 3 Lab of Systems Neuroscience, Department of Health Science and Technology, Institute for Neuroscience, Swiss Federal Institute of Technology ETH & Neuroscience Center Zurich (ZNZ), Zurich, Switzerland; 4 Institute for Physiological Chemistry, Philipps-University Marburg, Marburg, Germany; 5 Center for Lifespan Changes in Brain and Cognition (LCBC), Department of Psychology, University of Oslo, Oslo, Norway; 6 Department of Biomedicine, University of Basel, Basel, Switzerland; 7 Institute of Medical Genetics and Pathology, University Hospital Basel, Basel, Switzerland; 8 Department of Genetic Epidemiology in Psychiatry, Central Institute of Mental Health, Medical Faculty Mannheim, University of Heidelberg, Mannheim, Germany; 9 Psychiatric Center Nordbaden, Wiesloch, Germany; 10 Isar Amper Klinikum München Ost, kbo, Haar, Germany; 11 Department of Psychology, Clinical Psychology and Psychotherapy, Eberhard Karls University Tübingen, Tübingen, Germany; 12 Department of Child and Adolescent Psychiatry, Psychosomatics and Psychotherapy, University Hospital Essen, University of Duisburg-Essen, Duisburg, Germany; 13 Institute of Psychiatric Phenomics and Genomics (IPPG), University Hospital, LMU Munich, Munich, Germany; 14 Department of Psychiatry and Psychotherapy, University Medical Center Göttingen, Göttingen, Germany; 15 Center for Innovative Psychiatry and Psychotherapy Research, Central Institute of Mental Health, Medical Faculty Mannheim, University of Heidelberg, Mannheim, Germany; 16 Institute of Neuroscience and Medicine (INM-1), Research Center Jülich, Jülich, Germany; 17 Centre for Human Genetics, University of Marburg, Marburg, Germany; 18 Present Address: Clinic for Psychiatry and Psychotherapy, Eberhard Karls University Tübingen, Tübingen, Germany

**Keywords:** Genetics, Bipolar disorder

## Abstract

Bipolar disorder (BD) is a complex mood disorder with a strong genetic component. Recent studies suggest that microRNAs contribute to psychiatric disorder development. In BD, specific candidate microRNAs have been implicated, in particular *miR-137*, *miR-499a*, *miR-708*, *miR-1908* and *miR-2113*. The aim of the present study was to determine the contribution of these five microRNAs to BD development. For this purpose, we performed: (i) gene-based tests of the five microRNA coding genes, using data from a large genome-wide association study of BD; (ii) gene-set analyses of predicted, brain-expressed target genes of the five microRNAs; (iii) resequencing of the five microRNA coding genes in 960 BD patients and 960 controls and (iv) in silico and functional studies for selected variants. Gene-based tests revealed a significant association with BD for *MIR499A*, *MIR708*, *MIR1908* and *MIR2113*. Gene-set analyses revealed a significant enrichment of BD associations in the brain-expressed target genes of *miR-137* and *miR-499a-5p*. Resequencing identified 32 distinct rare variants (minor allele frequency < 1%), all of which showed a non-significant numerical overrepresentation in BD patients compared to controls (*p* = 0.214). Seven rare variants were identified in the predicted stem-loop sequences of *MIR499A* and *MIR2113*. These included rs142927919 in *MIR2113* (*p*_nom_ = 0.331) and rs140486571 in *MIR499A* (*p*_nom_ = 0.297). In silico analyses predicted that rs140486571 might alter the *miR-499a* secondary structure. Functional analyses showed that rs140486571 significantly affects *miR-499a* processing and expression. Our results suggest that *MIR499A* dysregulation might contribute to BD development. Further research is warranted to elucidate the contribution of the *MIR499A* regulated network to BD susceptibility.

## Introduction

Bipolar disorder (BD) is a common mental disorder that is characterized by recurrent episodes of depression and (hypo)mania, as well as mixed features and psychotic symptoms in some cases [1, 2]. The estimated lifetime prevalence of BD is around 1–2% [3, 4]. Notably, BD is associated with an increased risk of suicide [5] and major socio-economic disadvantage [6].

Early family and twin studies demonstrated that despite an estimated heritability of around 60–85% [7, 8], BD cannot be based on a simple Mendelian hereditary pattern. Rather, a consensus exists that BD involves an interaction between complex polygenic mechanisms and diverse environmental factors [9]. In recent years, genome-wide association studies (GWAS) have identified several common risk loci for BD (e.g. refs. [10–18]). The identified effects are modest or small and seem to accumulate predominantly in specific biological pathways (e.g. refs. [16, 18, 19]). Collectively, common variants account for around 20–30% of the total heritability of BD [16].

In recent years, several studies have investigated the contribution of rare variants in BD. The largest exome sequencing study to date was conducted by Palmer and colleagues [20] and comprised 13,933 patients with BD and 14,422 controls. However, in the primary gene-based analysis of this study, no gene showed an exome-wide significant association with BD.

MicroRNAs (miRNAs) are small, non-coding RNAs (~22 nucleotides) that act as important mediators of the epigenetic regulation of gene expression [21]. The canonical pathway of miRNA biogenesis commences with transcription from a DNA sequence into a primary (pri-) miRNA characterized by the presence of a typical stem-loop (or hairpin) structure, followed by a two-step processing via a precursor (pre-) into a single-stranded mature miRNA [21]. The first processing reaction is mediated by Drosha, a nuclear RNAse, which requires specific sequence elements in the stem-loop structure for efficient cleavage and production of pre-miRNA [22]. Pre-miRNAs are then further processed by the RNAse Dicer in the cytosol, generating an intermediary duplex miRNA [21]. From this intermediary duplex miRNA, one or both strands (designated 5p- or 3p-, depending on their original location within the pre-miRNA) are selected and separately incorporated into a microRNA-induced-silencing-complex (miRISC) [23]. As such, miRNAs bind specific target sequences in messenger RNAs (mRNAs), usually in their 3’ untranslated region (UTR), in order to induce mRNA degradation and translational inhibition [21]. However, additional target sites (5’UTR, coding sequences, promotor regions) and mechanisms of action (e.g. upregulation of gene expression, cell–cell interaction) have also been described [21]. Since a single miRNA can target several hundred mRNAs, and one mRNA can be controlled by several miRNAs [24], complex regulatory networks are created.

Research has shown that miRNAs have effects on synaptic function and plasticity, as well as on numerous neuronal development processes [25, 26]. A plausible hypothesis, therefore, is that miRNAs contribute to the development of neuropsychiatric diseases, including BD [27, 28].

Initial studies have shown significantly altered miRNA expression levels in the postmortem brain tissue of BD patients compared to controls [29, 30]. Analyses of the blood of BD patients have also shown alterations in circulating miRNAs [31], which suggests that miRNAs represent potential disease biomarkers.

One of the strongest findings of a large GWAS of BD [15] was a single-nucleotide polymorphism (SNP) in an intergenic region flanking *MIR2113*. Using the summary statistics of this BD GWAS, we performed a genome-wide analysis of miRNA coding genes [32]. This identified nine BD-associated miRNA coding genes, of which the brain-expressed *MIR499*, *MIR708* and *MIR1908* were considered the most promising candidates for further analysis [32]. Notably, a study by Banach and colleagues provided further evidence that these three miRNAs might be potential biomarkers for depressive episodes in BD patients [33]. Furthermore, a study by Strazisar et al. [34] identified two functional variants flanking *MIR137*, which were found more frequently in BD patients compared to controls. Interestingly, *MIR137* is located in a genome-wide significant risk locus for schizophrenia [35], a disorder that shows a strong genetic correlation with BD [36].

The aim of the present study was to determine the contribution of the five miRNAs encoded by *MIR137*, *MIR499A*, *MIR708*, *MIR1908* and *MIR2113* to the development of BD. For this purpose, a four-step investigation was conducted. This comprised gene-based tests of miRNA coding genes, gene-set analyses of predicted, brain-expressed miRNA target genes, resequencing of candidate miRNA coding genes and in silico and functional studies of selected variants.

## Materials and methods

### Gene-based tests of miRNA coding genes

Gene-based tests were performed using publicly available GWAS summary statistics from the BD Working Group of the Psychiatric Genomics Consortium (PGC), comprising data from 41,917 BD patients and 371,549 controls of European origin [16]. The gene-based tests were performed for candidate miRNA genes *MIR137*, *MIR499A*, *MIR708*, *MIR1908* and *MIR2113* (RefSeq definitions [37]) using the gene analysis implemented in MAGMA v1.09 (http://ctg.cncr.nl/software/magma; [38]). Briefly, SNPs within each predicted miRNA stem-loop sequence ±20 kilobases (kb) flanking sequences [32] were grouped together and analyzed using the SNP-wise Mean model. The generated *P*-values were Bonferroni-corrected for the number of tested miRNAs (*n* = 5).

### Gene-set analyses of brain-expressed target genes

Gene-set analyses were performed to test for enrichment of genes associated with BD in the predicted target genes of the five investigated miRNAs. For this purpose, the target genes for *miR-137*, *miR-499a-5p*, *miR-708-5p*, *miR-1908-5p* and *miR-2113* were retrieved from the TargetScan database (release 8.0; http://www.targetscan.org/vert_80/; [39]). The BrainSpan database (https://www.brainspan.org/; [40]) was accessed to determine whether the target genes were expressed in the human brain. Average expression values were calculated for six donors and nine different brain regions (amygdaloid complex, cerebellar cortex, dorsolateral prefrontal cortex, hippocampus, inferolateral temporal cortex, primary motor cortex, primary somatosensory cortex, posterior (caudal) superior temporal cortex and ventrolateral prefrontal cortex). Genes with a mean expression of >1.0 Reads Per Kilobase Million (RPKM) were considered brain-expressed. Gene-set analyses of the predicted, brain-expressed target genes were performed using summary statistics from the GWAS of the BD Working Group of the PGC [16] and MAGMA v1.09 [38]. SNPs were assigned to genes if they were located within the gene boundaries and ±20 kb flanking sequences. As an enrichment of BD associations has been reported for genes expressed in different brain tissues [16], brain-expressed genes [40] were used as the background for the gene-set analyses. The generated *P*-values were corrected for multiple testing using the Bonferroni method, taking into account the number of tested target gene networks (*n* = 5).

### Resequencing sample

The resequencing sample comprised 960 BD patients and 960 controls of German origin.

All 960 patients (44.8% male, 55.2% female) were diagnosed with BD according to DSM-IV criteria. Patient recruitment was carried out at the Central Institute of Mental Health in Mannheim, University of Heidelberg, and other collaborating psychiatric hospitals in Germany.

The controls (46.6% male, 53.4% female) were drawn from a population-based sample that was recruited in the Bonn area as part of the German National Genome Research Network [41].

The study was approved by the respective local ethics committees. All participants provided written informed consent.

### Resequencing

DNA was extracted from peripheral venous blood samples using standard methods. The chromosomal positions of *MIR137*, *MIR499A*, *MIR708*, *MIR1908* and *MIR2113* (RefSeq definitions [37]) were retrieved from the UCSC Genome Browser (assembly Feb. 2009, GRCh37/hg19). Primers were designed using the Primer3 software [42]. The resulting amplicons were between 337 and 694 base pairs (bp) in size, including the respective predicted miRNA stem-loop sequence and flanking sequences. Polymerase chain reactions (PCR) were carried out on Bio-Rad C1000 and Bio-Rad S1000 Thermal Cyclers (Bio-Rad, Hercules, CA, USA). Purification was performed using the AMPure XP PCR Purification kit and the Sanger Dye Terminator Removal kit of Beckman Coulter (Indianapolis, IN, USA). Sequence information was generated by Beckman Coulter Genomics (Essex, UK) on an Applied Biosystems PRISM 3730xl capillary sequencer (DNA Analyzer), and analyzed by the present authors using SeqMan II expert sequence analysis software (DNAStar, Madison, WI, USA). Variants were considered rare if they had a minor allele frequency (MAF) < 1% in the combined case-control sample, and novel if they were not present in the dbSNP 154 database [43]. All rare variants located in the predicted miRNA stem-loop sequence were validated via Sanger sequencing of an independent amplicon.

Primer sequences, further reagents used in experiments, and specific experimental conditions are available from the authors upon request. Due to experimental failures, individual miRNA coding genes and genetic variants were not investigated in all patients and controls. For quality control, a call rate of 95% (combined patients and controls) was applied for detected variants. One rare sequence variant near *MIR137* was excluded due to a low call rate (50.1%).

To evaluate the association with case-control status of common and rare sequence variants and the variable number tandem repeat (VNTR) at *MIR137*, Fisher’s exact tests (two-tailed) were performed in *R* v3.6.1, based on the distribution of allele counts. *P*-values for the different repeat alleles of the VNTR (*n* = 12) were corrected for multiple testing using the Bonferroni method.

Statistical evaluation of all rare variants at the five microRNA loci was also performed using Fisher’s exact test (two-tailed), taking into account the number of BD patients and controls who carried at least one rare sequence variant.

### Secondary structure prediction

Secondary structures were created using the Vienna RNA Package 2.0 [44] in order to determine whether the detected variants in *miR-499a* (rs140486571), *miR-2113* (rs142927919) and *miR-1908* (rs174561) led to changes in the predicted miRNA stem-loop structure present in the pre- and/or pri-miRNA sequence. Secondary structure prediction was performed using the minimum free energy (MFE) prediction algorithm.

### Functional analysis of selected variants

Functional analyses were performed for rs140486571 in *MIR499A* and rs174561 in *MIR1908*, since these variants were predicted to alter the secondary structure of the stem-loop present in the respective pre-/pri-miRNAs.

### DNA plasmids

Primary miRNAs were amplified from human genomic DNA using Pfu Plus! DNA Polymerase (Roboklon GmbH, Berlin, Germany), in accordance with the manufacturer’s instructions, and *XbaI-* and *SalI*-flanked primers. These primary miRNAs were then inserted into the pmirGLO dual-luciferase expression vector (Promega, Madison, WI, USA). Variants in *miR-499a* (rs140486571) and *miR-1908* (rs174561) were produced by site‐directed mutagenesis using Pfu Plus! DNA Polymerase (Roboklon GmbH, Berlin, Germany), in accordance with the manufacturer’s instructions.

shRNA sequences to silence the pri-miRNA processing enzyme Drosha and control shRNA were obtained from Dharmacon siRNA online design centre and cloned into pSuper vector (Oligoengine, Seattle, WA, USA) for transfections, as described elsewhere [45].

### Primer sequences (5’ to 3’)

#### Cloning

hsa-pri-miR-499a_Fwd: TGTCTCTAGACATCGTTCCAGACGGTGTCC

hsa-pri-miR-499a_Rev: TATAGTCGACGAATTGGATGCCGCAGTGGT

hsa-pri-miR-1908_Fwd: TGTATCTAGACCTATCCACTACCCTGGCG

hsa-pri-miR-1908_Rev: TATAGTCGACGGGCACTTCTGCGTTTCTTC

Control shRNA_Fwd 1: GATCCCCAAACCTTGTGGTCCTTAGGTTCAAGAGA

Control shRNA_Fwd 2: CCTAAGGACCACAAGGTTTTTTTTA

Control shRNA_Rev 1: AGCTTAAAAAAAACCTTGTGGTCCTTAGGTCTCTTGAA

Control shRNA_Rev 2: CCTAAGGACCACAAGGTTTGGG

Drosha shRNA_Fwd 1: GATCCCCCAACATAGACTACACGATTTTCAAGAGA

Drosha shRNA_Fwd 2: AATCGTGTAGTCTATGTTGTTTTTGGAAA

Drosha shRNA_Rev 1: AGCTTTTCCAAAAACAACATAGACTACACGATTTCTCTTGAA

Drosha shRNA_Rev 2: AATCGTGTAGTCTATGTTGGGG

#### Mutagenesis

Mutant hsa-pri-miR-499a_Fwd: GCCCTGTCCCCTGTGCCTTGGGCAGGCGGCTGTTAAGACTTGCAG

Mutant hsa-pri-miR-499a_Rev: CTGCAAGTCTTAACAGCCGCCTGCCCAAGGCACAGGGGACAGGGC

Mutant hsa-pri-miR-1908_Fwd: CCGCAGTGTGATTTGGGGCCGGGAATGCCGCGGCGGGGAC

Mutant hsa-pri-miR-1908_Rev: GTCCCCGCCGCGGCATTCCCGGCCCCAAATCACACTGCGG

### Primary neuronal cell culture

Primary hippocampal and cortical cultures were prepared from embryonic day 18 (E18) male and female Sprague Dawley rats (Charles River Laboratories, Sulzfeld, Germany), as described elsewhere [46]. Hippocampal neurons were plated on acid-treated coverslips coated with Poly-L-Lysine (Sigma, Steinheim, Germany) and Laminin (BD Biosciences, San Jose, CA, USA). Cortical neurons were directly cultured on plates coated with Poly-L-Ornithine (Sigma). Neurons were maintained in Neurobasal medium containing 2% B-27 supplement, 2 mM GlutaMAX, 100 U/ml penicillin and 100 μg/ml streptomycin (Gibco, Thermo Fisher Scientific, Waltham, MA, USA) in a humidified incubator with 5% CO_2_ at 37 °C.

### Transfections

For luciferase assays, hippocampal neurons were plated at a density of 75,000 cells per well on a 24-well plate. Cells were transfected at *DIV* 15 with 100 ng of wild-type or mutant pmirGLO luciferase reporters and 5 ng of shRNAs using the Lipofectamine 2000 reagent (Invitrogen, Thermo Fisher Scientific, Waltham, MA, USA), in accordance with the manufacturer’s instructions. For quantitative PCR (qPCR), four million freshly isolated cortical neurons were transfected with 2 µg of wild-type or mutant pmirGLO luciferase reporters using the P3 Primary Cell 4D-Nucleofector Kit (Lonza, Basel, Switzerland), in accordance with the manufacturer’s instructions.

### HEK293T cell culture and transfection

HEK293T cells were obtained from the American Type Culture Collection (ATCC) and regularly checked for mycoplasma contamination using PCR-based assays. HEK293T cells were maintained in DMEM supplemented with 10% fetal bovine serum (FBS), 1 mM glutamine, 100 U/ml penicillin and 100 μg/ml streptomycin (Thermo Fisher Scientific, Waltham, MA, USA), in a humidified incubator with 5% CO_2_ at 37 °C. One day prior to transfection, cells were seeded in 24-well plates for luciferase reporter assay experiments or six-well plates for qPCR. HEK293T cells were transfected with 0.6 μg of DNA per well of a 24-well plate and 2.4 μg per well of a six-well plate using the calcium phosphate method, with a final CaCl_2_ concentration of 0.1 M.

### Luciferase reporter assay

Neurons and HEK293T cells were lysed in 5× Passive Lysis Buffer (Promega) 72 h post-transfection. Luciferase assay experiments were performed using a modified Dual-Luciferase Reporter Assay System, as described previously by Baker & Boyce [47], and the GloMax R96 Microplate Luminomiter (E1941, Promega). Relative luciferase activity was calculated as a ratio of Firefly to Renilla signal.

### Quantitative real-time PCR

Total RNA was isolated using the mirVana miRNA isolation kit (AM1561, Invitrogen, Thermo Fisher Scientific, Waltham, MA, USA), in accordance with the manufacturer’s instructions, and then treated with TURBO^™^ DNase (Ambion, Thermo Fisher Scientific, Waltham, MA, USA) to remove potential genomic DNA contaminations. RNA was reverse transcribed using the TaqMan MicroRNA Reverse Transcription kit (4366597, Applied Biosystems, Thermo Fisher Scientific, Waltham, MA, USA), in accordance with the manufacturer’s protocols. Quantitative real-time PCR was performed with the StepOnePlus Real‐Time PCR System (Applied Biosystems, Thermo Fisher Scientific, Waltham, MA, USA), and TaqMan Universal PCR Master Mix (4364341, Applied Biosystems, Thermo Fisher Scientific, Waltham, MA, USA). For the quantification of mature *miR-499a*, TaqMan miRNA Assays hsa‐miR-499a‐5p (Assay ID: 001352) and U6 snRNA (Assay ID: 001973) were used.

### Northern Blot

Northern Blot detection of mature and precursor *miR-499a* was performed as described elsewhere [48]. HEK293T cells were seeded in six-well plates and transfected with 250 ng of pmirGLO plasmids containing either pri-miR-499a wild-type or the rs140486571 variant. Twenty micrograms of total RNA were separated in a denaturing urea 15% PAGE gel (Mini-PROTEAN system; Bio-Rad, Hercules, CA, USA), and blotted onto a GeneScreen Plus nylon membrane (PerkinElmer, Waltham, MA, USA) using radioactively labelled Decade marker (Ambion, Thermo Fisher Scientific, Waltham, MA, USA) as a molecular marker. RNA was crosslinked to the membrane by ultraviolet irradiation (1200 mJ), followed by baking of the membrane for 30 min at 80 °C. The membrane was pre-incubated in hybridization buffer (5 × SSC, 20 mM Na_2_HPO_4_ (pH = 7.2), 7% SDS, 2 × Denhardt’s solution, 40 μg/mL salmon sperm DNA) for at least 2 h at 50 °C at constant rotation, followed by incubation overnight at 50 °C in hybridization buffer containing the denatured [32 P] labelled DNA probe. The membrane was washed twice for 10 min and twice for 30 min at 50 °C with a non-stringent wash solution (3 × SSC, 25 mM NaH_2_PO_4_ (pH = 7.5), 5% SDS, 10 × Denhardt’s solution), and then once for 5 min at 50 °C with stringent wash solution (1 × SSC, 1% SDS). RNA detection was conducted via autoradiography using the Cyclone Plus Phosphor Imager (PerkinElmer, Waltham, MA, USA). The DNA probes were as follows (5’ to 3’):

*U6 snRNA:* GCAGGGGCCATGCTAATCTTCTCTGTATCG

*miR-499a:* TGAACATCACAGCAAGTCTGT

### Statistics

Data are represented as means ± standard deviation. Three independent experiments were performed for each dataset, unless otherwise specified. Normal distribution was tested with the D’Agostino & Pearson omnibus normality test, the Shapiro-Wilk normality test and the KS normality test. *P*-values were calculated using the unpaired Student’s *t*-test (two-tailed, heteroscedastic) for one-way comparisons, or two-way ANOVA followed by Tukey’s post hoc test for multi-way comparisons. Data were analyzed using GraphPad Prism. In all graphs, the significance level is indicated by * for *p* < 0.05, ** for *p* < 0.01 and *** for *p* < 0.001.

## Results

### Gene-based tests of miRNA coding genes

A significant association was found for the miRNA coding genes *MIR499A*, *MIR708*, *MIR1908* and *MIR2113* after correction for multiple testing (Table 1). Of these, the strongest association with BD was found for *MIR1908* (*p*_corr_ = 5.38 × 10^−12^). No association with BD was found for *MIR137* (*p*_corr_ = 0.697).

**Table 1:** Results of the gene-based tests for the five microRNA coding genes.

GENE	CHR	START	STOP	NSNPS	NPARAM	ZSTAT	*P*	*P*_corr_
*MIR137*	1	98491626	98531727	111	10	1.0829	0.139	0.697
***MIR2113***	6	98452407	98492497	64	11	4.6726	1.49E-06	**7.44E-06**
***MIR1908***	11	61562633	61602712	52	6	7.0242	1.08E-12	**5.38E-12**
***MIR708***	11	79093066	79133153	147	13	3.3019	4.80E-04	**0.002**
***MIR499A***	20	33558179	33598300	101	11	2.3858	0.009	**0.043**

Overview of the results of the gene-based tests for the five microRNA (miRNA) coding genes, as performed using MAGMA [38] and the summary statistics of a large bipolar disorder (BD) genome-wide association study [16]. Chromosomal positions are given according to hg19. The miRNA coding genes indicated in bold font showed a significant association with BD after Bonferroni correction for multiple testing.

*CHR* chromosome, *NSNPS* number of single-nucleotide polymorphisms, *NPARAM* number of parameters used in the model, *ZSTAT* z-value of the miRNA coding gene, *P* *P*-value of the miRNA coding gene, *P*_corr_ *P*-value after Bonferroni correction for multiple testing.

### Gene-set analyses of brain-expressed target genes

The gene-set analyses revealed a significant enrichment of BD associations in the predicted, brain-expressed target genes of *miR-137* and *miR-499a-5p* after Bonferroni correction for multiple testing (Table 2).

**Table 2:** Results of the gene-set analyses of brain-expressed target genes of the five microRNAs.

MIRNA	NGENES	BETA	BETA_STD	SE	*P*	*P*_corr_
***miR-137***	1051	0.1194	0.0346	0.0366	5.53E-04	**0.003**
*miR-2113*	3102	0.0533	0.0237	0.0234	0.011	0.057
*miR-1908-5p*	1730	0.0650	0.0234	0.0287	0.012	0.059
*miR-708-5p*	70	0.0594	0.0046	0.1217	0.313	>0.999
***miR-499a-5p***	344	0.1488	0.0255	0.0610	0.007	**0.037**

Overview of the results of the gene-set analyses of the predicted, brain-expressed target genes of the five microRNAs (miRNAs), as performed using MAGMA [38] and the summary statistics of a large bipolar disorder (BD) genome-wide association study [16]. A significant enrichment of BD associations was detected for the brain-expressed target genes of two of the five miRNAs (indicated in bold font) after Bonferroni correction for multiple testing.

*NGENES* number of predicted brain-expressed target genes [39, 40], *BETA* regression coefficient of the target gene-set, *BETA_STD* semi-standardized regression coefficient, *SE* standard error of the regression coefficient, *P* *P*-value of the brain-expressed target genes of the miRNA, *P*_corr_ *P*-value after Bonferroni correction for multiple testing.

### Resequencing

A total of 36 independent sequence variants were identified at the five miRNA loci. These included four common variants (MAF ≥ 1%; rs174561 in *MIR1908*, rs3746444 in *MIR499A*, rs117428639 in *MIR2113* and rs9375085 downstream of *MIR2113*). None of these four common variants showed an association with BD in the resequencing sample. The lowest nominal *P*-value in the resequencing step was found for rs174561 (*p*_nom_ = 0.053).

The 32 distinct rare sequence variants (MAF < 1%, Supplementary Table 1) showed a non-significant numerical overrepresentation in BD patients compared to controls (*n* = 57 in 57 patients versus *n* = 42 in 42 controls, *p* = 0.214; Table 3). The rare variants comprised single-nucleotide substitutions, as well as small deletions and insertions. All of the observed rare variants were found in a heterozygous state.

**Table 3:** MicroRNA-based distribution of the identified rare sequence variants.

Gene	All variants		Variants located in the predicted microRNA stem-loop sequence	
	Patients	Controls	Patients	Controls
*MIR2113*	26	18	11	7
*MIR499A*	24	14	11	8
*MIR137*	3	3	0	0
*MIR708*	3	3	0	0
*MIR1908*	1	4	0	0
Total	57	42	22	15

Minor allele frequency of variants <1%; patients = number of alterations observed in patients; controls = number of alterations observed in controls.

Seven rare variants were identified within the predicted stem-loop sequences of *MIR499A* and *MIR2113*. These included a rare guanosine to adenosine substitution (rs142927919) in *MIR2113*, which was detected in 10 BD patients (four males, six females; mean age at onset according to the Operational Criteria Checklist for Psychotic Illness and Affective Illness (OPCRIT): 26.4 years) and six controls (two males, four females; *p*_nom_ = 0.331). In *MIR499A*, a rare point mutation (rs140486571) was detected in nine BD patients (three males, six females; mean age at onset according to OPCRIT: 31.6 years) and five controls (all male; *p*_nom_ = 0.297). No rare variants were detected in the predicted stem-loop sequences of *MIR137*, *MIR708*, or *MIR1908*.

At the *MIR137* locus, investigations were performed into a 15 bp long VNTR (rs58335419; Supplementary Table 2). In the resequencing sample, 3–14 repeats of the VNTR were observed. Of the various repeat alleles, only the 11 repeat allele showed a nominally significant overrepresentation in BD patients compared to controls (*p*_nom_ = 0.009). However, this association did not withstand stringent Bonferroni correction for multiple testing (*p*_corr_ = 0.103).

### Secondary structure prediction

**Fig. 1:**
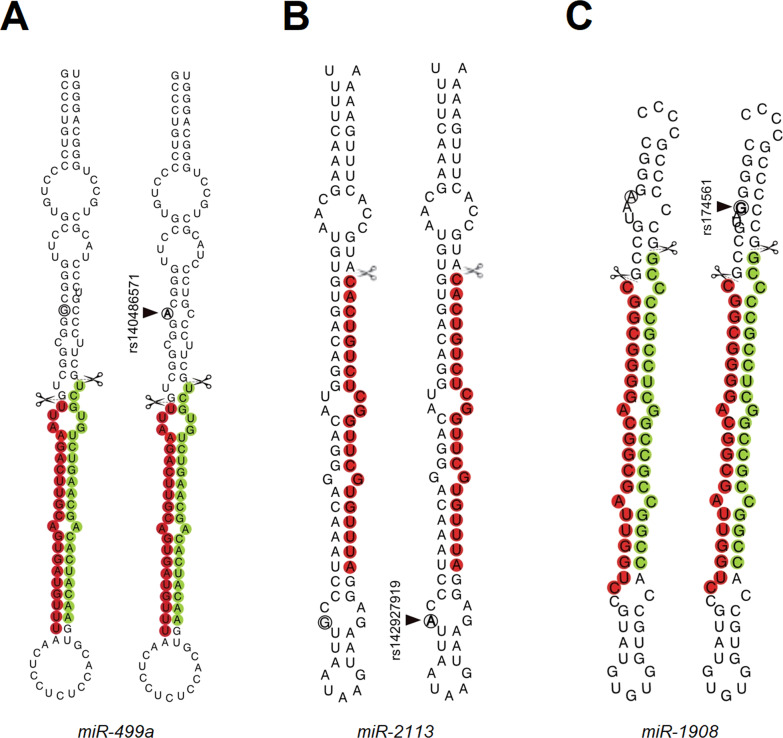
Secondary structure prediction. Minimum free energy (MFE) secondary structure prediction of **A** *miR-499a* wild-type and rs140486571 (G/A); **B** *miR-2113* wild-type and rs142927919 (G/A) and **C** *miR-1908* wild-type and rs174561 (A/G) sequences. The positions of the variant nucleotides are indicated by black circles. Regions of the mature miRNAs are highlighted in colour (green for 3p, red for 5p). Drosha cleavage sites on both strands are indicated by scissors.

Secondary structure predictions were performed for rs140486571 in *MIR499A*, rs142927919 in *MIR2113* and rs174561 in *MIR1908* (Fig. 1). For rs140486571 and rs174561, minor changes in the predicted secondary structure were observed for the predicted stem-loops of *miR-499a* and *miR-1908* compared to the respective wild-type sequences. Although these did not affect the mature miRNA sequence, they were located in close proximity to the predicted Drosha cleavage sites (Fig. 1A, C). For rs142927919, no change in the secondary structure of *miR-2113* was observed (Fig. 1B).

### Functional analyses

Functional analyses were performed for rs140486571 in *MIR499A* and rs174561 in *MIR1908*, since these variants were predicted to alter the secondary structure of the respective miRNAs.

To assess the functional relevance of the two variants in terms of the biogenesis of the respective miRNAs, luciferase reporter assays were performed in HEK293T cells and primary rat hippocampal neurons. Either wild-type or mutant (rs140486571/miR-499a; rs174561/miR-1908) pri-miRNA stem-loop sequences were cloned downstream of the luciferase coding sequence (cds) within the pmiR-Glo dual luciferase vector. Upon transfection into cells, enhanced or impaired pri-miRNA processing is expected to result in reduced or elevated luciferase expression, respectively.

**Fig. 2:**
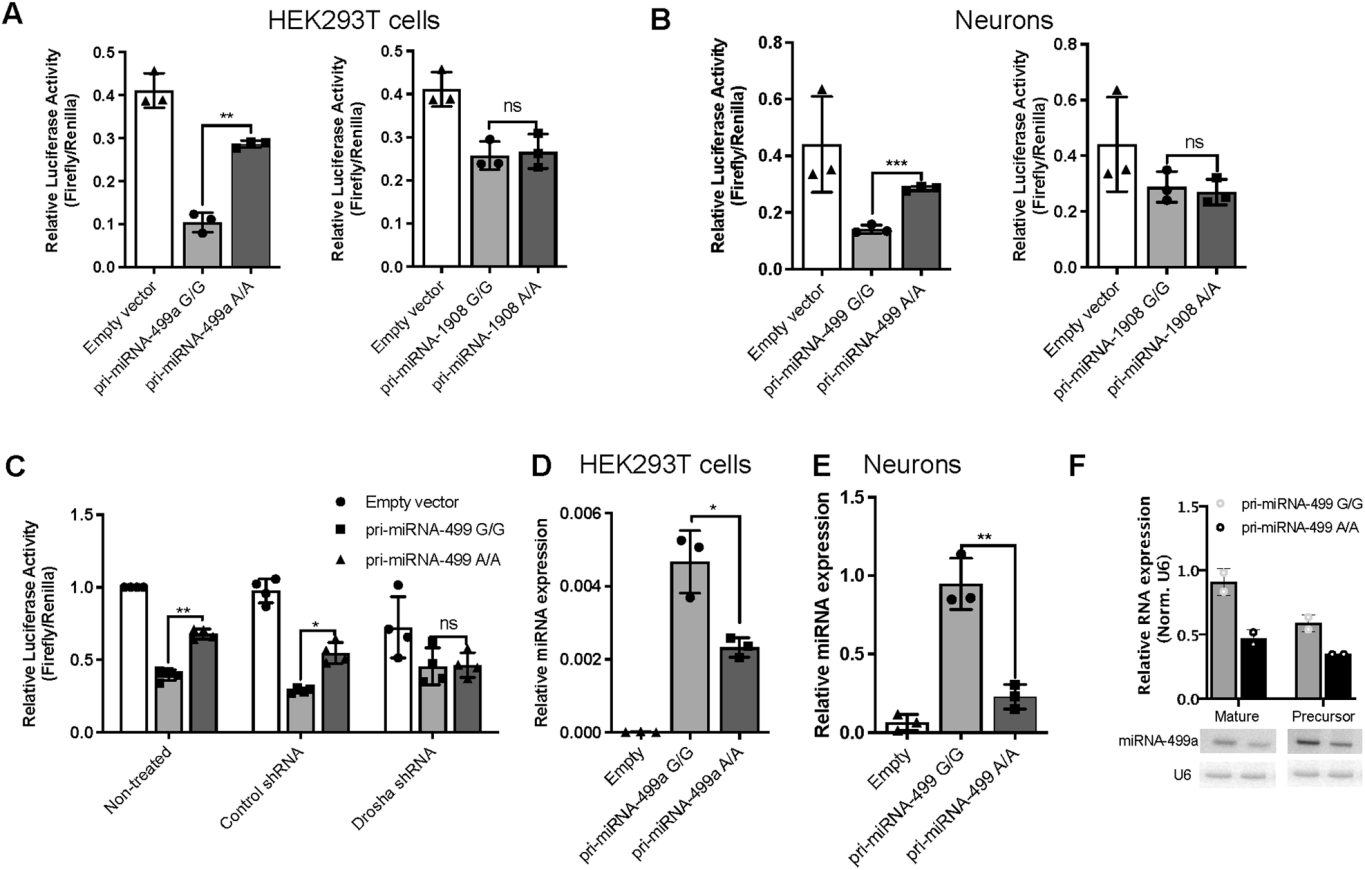
Variant rs140486571 significantly affects *miR-499a* processing and expression. miRNA processing was evaluated using luciferase reporter assay experiments in HEK cells (**A**) and hippocampal neurons (**B**) transfected with pmirGLO plasmids. The plasmids contained either a wild-type sequence, or a sequence bearing the variant of pri-miR-499a (pri-miR-499a G/G and A/A, respectively) or pri-miR-1908 (pri-miR-1908 A/A and G/G, respectively). Relative luciferase activity represents the ratio of Firefly to Renilla control reporter activity. **C** Luciferase reporter assay from hippocampal neurons transfected with pri-miR-499a-G/G and A/A, both with and without Drosha knockdown directed by an shRNA (shDrosha), demonstrates that Drosha is unable to process the mutant *miR-499* variant. Relative levels of mature *miR-499* were assessed using qPCR in HEK cells (**D**) and cortical neurons (**E**) transfected with pri-miR-499a G/G or A/A. Data represent relative *miR-499a* expression, normalized to U6 snRNA expression (2^−ΔCt^). **F** Northern blot analysis of *miR-499a* and pre-miR-499a in HEK cells transfected with pri-miR-499a G/G and A/A. U6 snRNA was probed and used as a control RNA. Data are presented as individual data points, with bar plots showing the mean and standard deviation (*n* = 3 independent experiments for luciferase reporter assays and qPCR, and *n* = 2 independent experiments for northern blot). Statistical significance between pri-miR-499a G/G and pri-miR-499a A/A groups was determined using an unpaired Student’s *t*-test (*p < 0.05, **p < 0.01, ***p < 0.001). ns not significant.

In both HEK293T cells and primary neurons (Fig. 2A, B), transfection of mutant *miR-499a* vector caused a significant increase in luciferase activity compared to the wild-type variant, indicating a strong impairment of the processing of the pri-miR-499a stem-loop sequence as a result of the G/A variant. In contrast, no differences were observed between the *miR-1908* mutant and wild-type vectors. To generate further insights into the mechanism underlying this impaired *miR-499a* processing, luciferase assay experiments were performed in the context of shRNA-directed Drosha knockdown (Fig. 2C). Drosha is the core enzyme of the microprocessor complex, and is essential for the efficient processing of pri-miRNAs into pre-miRNAs. Drosha knockdown selectively elevated the luciferase activity of the wild-type, but not the mutant *miR-499a* vector, demonstrating that Drosha is responsible for the differences in processing efficiencies between the wild-type and G/A variant. Analyses to determine whether impaired *miR-499a* processing translated into reduced levels of mature *miR-499a* revealed that transfection of the mutant *miR-499a* construct led to significantly less mature miRNA expression compared to the wild-type in both HEK293T cells (Fig. 2D) and primary rat cortical neurons (Fig. 2E), as determined by qPCR. Similarly, Northern blot experiments (Fig. 2F) showed a trend towards a reduction in the levels of mature *miR-499a* as well as pre-miR-499a in HEK293T cells transfected with the mutant *miR-499a* construct, which is consistent with impaired Drosha-mediated pri-miR-499a processing secondary to the mutation.

## Discussion

In the present study, a systematic investigation was performed to determine the contribution of five candidate miRNAs to the development of BD.

The gene-based tests revealed a significant association with BD for the miRNA coding genes *MIR499A*, *MIR708*, *MIR1908* and *MIR2113* after stringent Bonferroni correction for multiple testing. These results are consistent with the findings of a previous study by our group [32], which showed significant associations with BD for *MIR499*, *MIR708* and *MIR1908* after correction for multiple testing, while the result for *MIR2113* was only nominally significant. In our previous investigation, the gene-based tests were performed using an alternative method (set-based testing approach adapted from the versatile gene-based test for GWAS [49]), and a smaller BD GWAS dataset (9747 patients, 14,278 controls [15]). Almost all of the latter were included in the larger PGC BD GWAS [16] investigated in the present study, which comprised a total of 41,917 patients and 371,549 controls.

The results of the gene-set analyses showed that genes with BD associations are significantly enriched in the predicted, brain-expressed target genes of the miRNAs 137 and 499A. These results provide further evidence that the network regulated by *MIR499A* is involved in the development of BD at both the miRNA coding gene- and the target gene level.

Interestingly, while enrichment was observed in the brain-expressed target genes of *miR-137*, no association between *MIR137* per se and BD was found in the gene-based tests for common genetic variants. Given that previous studies reported a genome-wide association between common variants in an intron of a putative primary transcript for *MIR137* and schizophrenia (e.g. ref. [50]), this finding may suggest that the contribution of the *MIR137* regulated network to disease development differs between schizophrenia and BD [36].

In the resequencing step, rare variants were identified in the predicted stem-loop sequences of *MIR2113* and *MIR499A* only. Of these, two variants (rs142927919 in *MIR2113* and rs140486571 in *MIR499A*) were found in more than two subjects and showed a statistically non-significant overrepresentation in BD patients compared to controls. In silico analyses predicted that while rs140486571 might affect the secondary structure of *miR-499a*, rs142927919 does not affect the secondary structure of *miR-2113*. Subsequent functional analyses generated evidence that rs140486571 significantly affects *miR-499a* processing and expression, which might lead to a dysregulation of downstream target gene networks. This, in turn, might contribute to the development of BD. Given the close proximity of rs140486571 to the predicted Drosha cleavage site (Fig. 1A), a plausible hypothesis is that reduced *miR-499a* expression is a result of impaired pri-miRNA processing by Drosha. The observation that differences in processing activity were occluded in Drosha knockdown cells (Fig. 2C) is consistent with this interpretation. Interestingly, although the common variant rs174561 also affected the secondary structure of *miR-1908* close to the Drosha cleavage site, this had no functional implications (Fig. 2A). Besides the distances from apical and basal junctions and cis-acting elements, internal loops and bulges can promote or hinder the interaction between pri-miRNA and Drosha [51, 52]. We, therefore, hypothesize that by adding a new bulge to the secondary structure of *miR-499a*, rs140486571 creates a physical barrier to proper recognition and/or binding of the microprocessor that is not observed in rs174561 (Fig. 1A, C).

With regard to molecular mechanisms, recent studies found that *miR-499* targets *CACNB2*, a regulatory subunit of L-type voltage-gated calcium channels (LVGCC), which are the primary mediators of depolarization-induced calcium entry into neurons [53, 54]. *CACNB2* is a reported susceptibility gene for BD [16]. Furthermore, calcium dysregulation—in particular of LVGCCs—has been frequently implicated in the pathogenesis of neuropsychiatric disorders, including BD [55]. A plausible hypothesis is that in rs140486571 carriers, *miR-499a* deficiency leads to elevated CACNB2 levels, which results in turn in increased intracellular calcium levels and BD susceptibility. This is supported by a recent meta-analysis of 21 independent studies, which found that the peripheral cells of BD patients showed increased levels of intracellular calcium [56]. Further experiments are warranted to investigate whether the miR-499/CACNB2 interaction is implicated in the pathogenesis of BD.

A previous study by Banigan et al. [29] demonstrated an alteration in the expression of exosomal *miR-499* in the post-mortem brains of BD patients compared to controls. Furthermore, in their investigation of BD patients, Banach and colleagues reported lower expression levels of *miR-499* during depressive episodes compared to the remission state [33], which might suggest that *miR-499* is a promising biomarker for depressive episodes in BD. In a previous study of lithium response in BD, the present authors performed a genome-wide analysis of miRNA coding genes and demonstrated a nominally significant association with *MIR499A* [28]. These findings, and the results of the present study, indicate that *MIR499A* might be involved in the development, disease course and treatment response of BD. Elucidation of the precise role of *MIR499A* in BD will require: (i) functional studies of the associated common genetic variants (e.g. miRNA expression quantitative trait loci (miR-QTL) studies) in relevant cells/tissues; and (ii) follow-up analyses of rare *MIR499A* variants in larger BD case-control samples.

The in silico secondary structure prediction for rs142927919 revealed no changes in *miR-2113*. However, this does not exclude the possibility that this variant has a functional effect. Therefore, rs142927919 should also be followed up via functional investigations and analyses in larger, independent sequencing cohorts.

In the other three miRNA coding genes (*MIR137*, *MIR708* and *MIR1908*), no rare variants were identified in the predicted stem-loop sequences. Although we cannot rule out the presence of ultra-rare sequence variants, the present results indicate that these genomic regions are highly conserved. If so, a plausible hypothesis is that genetic changes in the mature miRNA sequences lead to more pronounced effects [57]. This might also explain the generally high conservation of miRNA sequences that has been demonstrated in previous studies [57–59].

The present results were compared with those of previous studies [34, 60, 61]. In an investigation of BD and schizophrenia, Fiorentino and colleagues reported three variants at the *MIR708* locus, i.e. rs754333774, rs768049399 and rs56158925 [61]. Of these, only rs754333774 was detected in our resequencing sample. However, the variant was not overrepresented in BD patients (one BD patient versus two controls, Supplementary Table 1). Variant rs768049399 was not detected in our sample, which might reflect the very low reported MAF of this variant. No statement can be made concerning variant rs56158925, since this chromosomal position was not covered by the selected primer pair.

At the *MIR137* locus, the -4C/T variant rs185304769 reported by Strazisar et al. [34] was identified in one patient and three controls from the present resequencing sample (Supplementary Table 1). Thus, the observation by Strazisar et al. [34] that this variant was overrepresented in patients with BD compared to controls was not replicated. With respect to the VNTR, the present analyses identified a nominally significant association of the 11-repeat allele with BD that did not withstand Bonferroni correction for multiple testing. As in the investigation of Strazisar et al. [34], the 8-repeat allele had a slightly higher frequency in BD patients compared to controls in the present sample (Supplementary Table 2). However, in contrast to Strazisar et al. [34], this overrepresentation was not statistically significant (*p*_corr_ > 0.999).

The rare BD-associated variant “1:g.98515539 A > T” reported by Duan et al. [60] is located around 3800 bp upstream of the precursor *MIR137*. This variant was not investigated in the present study, since the resequencing approach focused on the predicted *MIR137* stem-loop sequence. Therefore, our results do not exclude the possibility that rare variants in regulatory elements (e.g. enhancers) flanking *MIR137* might contribute to BD development.

In addition, we compared our results with data from the BipEx browser (https://bipex.broadinstitute.org/), which includes findings from the recent Palmer et al. study [20]. The BipEx browser listed variants at the loci of miRNAs 137 and 499 A (as of July 2021). At the *MIR137* locus, the variant rs185304769 was also detected in BipEx at a slightly higher frequency in controls compared to BD patients (MAF = 6.12E-04 in BD patients versus 8.34E-04 in controls, *p* = 0.406). At the *MIR499A* locus, four variants identified in the present study were also listed in the BipEx browser. The variant rs557768162 was identified in one patient with BD and no controls. The other three variants rs140486571, rs150018420 and rs7267163 were found in nearly comparable frequencies in patients and controls, and showed no association with BD (*p* > 0.684 [20]). These results provide no strong evidence for an association between BD and the variant rs140486571, which showed functional effects on *miR-499a* processing and expression in the present analyses. However, the possibility that rs140486571 contributes to BD development with a small effect size is not completely excluded. Therefore, follow-up studies of this variant in larger case-control cohorts are required before definitive conclusions can be drawn.

The present study had several limitations. First, the gene-based tests investigated variants in the predicted stem-loop sequences of miRNA coding genes as well as in flanking sequences. However, four of the five analyzed miRNAs (i.e. miRNAs 137, 499A, 708 and 1908) are located in host genes. Therefore, the applied statistical approach cannot determine whether the associations detected by the gene-based tests refer to the miRNA, the host gene, both the miRNA and the host gene, or to nearby functional elements [32]. To determine their relevance, functional analyses of the BD-associated genetic variants are therefore necessary, e.g. experiments involving the use of miR-QTL data. Second, the gene-set analyses were focused on brain-expressed target genes, since an enrichment of BD associations has been reported for genes expressed in different brain tissues [16]. However, it should be noted that a prerequisite for the interaction between miRNAs and target genes is their co-expression in tissues or cells. Future studies should therefore investigate the tissue- and cell-specific regulation of the predicted target gene networks by the respective miRNA in order to identify those target genes that are (most) relevant for BD development. Third, although the present study investigated almost 1000 BD patients and 1000 controls, the power to detect associations with rare variants was limited [62]. Fourth, the present study focused on five miRNAs reported in the literature. However, our previous genome-wide analysis of common variants [32] showed that miRNA loci, in general, are enriched for associations with BD. Systematic investigations are therefore warranted to determine the contribution of rare variants at all known miRNA loci, e.g. via targeted (re-)analysis of whole-genome sequencing data, or by applying innovative next-generation sequencing technologies, such as molecular inversion probes [63].

Given that the regulation of miRNA function is highly complex, a lack of evidence of BD-associated common or rare sequence variants at the miRNA locus does not exclude the involvement of the respective miRNA-regulated network in disease development. Among others, relevant variants have been identified in the 3’UTRs of target genes [64] or in nearby located regulatory elements [60]. The present analyses revealed significant enrichment for the brain-expressed *MIR137* target gene network, while common variants at the *MIR137* locus were not associated with BD in the gene-based tests. However, future systematic research is required to determine the contribution of common and rare genetic variants at the various genomic levels of miRNA-regulated networks.

In conclusion, the results of the present genetic and functional analyses provide further evidence that *MIR499A* may be involved in the development of BD. Further research is warranted to elucidate the molecular network that is regulated by *MIR499A*, and its complex contribution to BD susceptibility.

## Supplementary information

Supplementary Table 1

Supplementary Table 2

## References

[1] American Psychiatric Association. Diagnostic and statistical manual of mental disorders (5th edn.). Arlington: American Psychiatric Association; 2013.

[2] Gordovez FJA, McMahon FJ (2020). The genetics of bipolar disorder. Mol Psychiatry.

[3] Merikangas KR, Akiskal HS, Angst J, Greenberg PE, Hirschfeld RM, Petukhova M (2007). Lifetime and 12-month prevalence of bipolar spectrum disorder in the National Comorbidity Survey replication. Arch Gen Psychiatry.

[4] Merikangas KR, Jin R, He JP, Kessler RC, Lee S, Sampson NA (2011). Prevalence and correlates of bipolar spectrum disorder in the world mental health survey initiative. Arch Gen Psychiatry.

[5] Pompili M, Gonda X, Serafini G, Innamorati M, Sher L, Amore M (2013). Epidemiology of suicide in bipolar disorders: a systematic review of the literature. Bipolar Disord.

[6] Jin H, McCrone P (2015). Cost-of-illness studies for bipolar disorder: systematic review of international studies. PharmacoEconomics.

[7] Bienvenu OJ, Davydow DS, Kendler KS (2011). Psychiatric ‘diseases’ versus behavioral disorders and degree of genetic influence. Psychol Med.

[8] Lichtenstein P, Yip BH, Bjork C, Pawitan Y, Cannon TD, Sullivan PF (2009). Common genetic determinants of schizophrenia and bipolar disorder in Swedish families: a population-based study. Lancet.

[9] Craddock N, Sklar P (2013). Genetics of bipolar disorder. Lancet.

[10] Charney AW, Ruderfer DM, Stahl EA, Moran JL, Chambert K, Belliveau RA (2017). Evidence for genetic heterogeneity between clinical subtypes of bipolar disorder. Transl Psychiatry.

[11] Chen DT, Jiang X, Akula N, Shugart YY, Wendland JR, Steele CJ (2013). Genome-wide association study meta-analysis of European and Asian-ancestry samples identifies three novel loci associated with bipolar disorder. Mol Psychiatry.

[12] Green EK, Grozeva D, Forty L, Gordon-Smith K, Russell E, Farmer A (2013). Association at SYNE1 in both bipolar disorder and recurrent major depression. Mol Psychiatry.

[13] Green EK, Hamshere M, Forty L, Gordon-Smith K, Fraser C, Russell E (2013). Replication of bipolar disorder susceptibility alleles and identification of two novel genome-wide significant associations in a new bipolar disorder case-control sample. Mol Psychiatry.

[14] Hou L, Bergen SE, Akula N, Song J, Hultman CM, Landen M (2016). Genome-wide association study of 40,000 individuals identifies two novel loci associated with bipolar disorder. Hum Mol Genet.

[15] Muhleisen TW, Leber M, Schulze TG, Strohmaier J, Degenhardt F, Treutlein J (2014). Genome-wide association study reveals two new risk loci for bipolar disorder. Nat Commun.

[16] Mullins N, Forstner AJ, O’Connell KS, Coombes B, Coleman JRI, Qiao Z (2021). Genome-wide association study of more than 40,000 bipolar disorder cases provides new insights into the underlying biology. Nat Genet.

[17] Psychiatric GWAS Consortium Bipolar Disorder Working Group. (2011). Large-scale genome-wide association analysis of bipolar disorder identifies a new susceptibility locus near ODZ4. Nat Genet.

[18] Stahl EA, Breen G, Forstner AJ, McQuillin A, Ripke S, Trubetskoy V (2019). Genome-wide association study identifies 30 loci associated with bipolar disorder. Nat Genet.

[19] Nurnberger JI, Koller DL, Jung J, Edenberg HJ, Foroud T, Guella I (2014). Identification of pathways for bipolar disorder: a meta-analysis. JAMA Psychiatry.

[20] Palmer DS, Howrigan DP, Chapman SB, Adolfsson R, Bass N, Blackwood D (2022). Exome sequencing in bipolar disorder identifies AKAP11 as a risk gene shared with schizophrenia. Nat Genet.

[21] O’Brien J, Hayder H, Zayed Y, Peng C (2018). Overview of microRNA biogenesis, mechanisms of actions, and circulation. Front Endocrinol.

[22] Gregory RI, Yan KP, Amuthan G, Chendrimada T, Doratotaj B, Cooch N (2004). The microprocessor complex mediates the genesis of microRNAs. Nature.

[23] Meijer HA, Smith EM, Bushell M (2014). Regulation of miRNA strand selection: follow the leader?. Biochem Soc Trans.

[24] Guo L, Zhao Y, Yang S, Zhang H, Chen F (2014). Integrative analysis of miRNA-mRNA and miRNA-miRNA interactions. BioMed Res Int.

[25] Geaghan M, Cairns MJ (2015). MicroRNA and posttranscriptional dysregulation in psychiatry. Biol Psychiatry.

[26] Schratt G (2009). microRNAs at the synapse. Nat Rev Neurosci.

[27] Martins HC, Schratt G (2021). MicroRNA-dependent control of neuroplasticity in affective disorders. Transl Psychiatry.

[28] Reinbold CS, Forstner AJ, Hecker J, Fullerton JM, Hoffmann P, Hou L (2018). Analysis of the influence of microRNAs in lithium response in bipolar disorder. Front Psychiatry.

[29] Banigan MG, Kao PF, Kozubek JA, Winslow AR, Medina J, Costa J (2013). Differential expression of exosomal microRNAs in prefrontal cortices of schizophrenia and bipolar disorder patients. PLoS ONE.

[30] Bavamian S, Mellios N, Lalonde J, Fass DM, Wang J, Sheridan SD (2015). Dysregulation of miR-34a links neuronal development to genetic risk factors for bipolar disorder. Mol Psychiatry.

[31] Maffioletti E, Tardito D, Gennarelli M, Bocchio-Chiavetto L (2014). Micro spies from the brain to the periphery: new clues from studies on microRNAs in neuropsychiatric disorders. Front Cell Neurosci.

[32] Forstner AJ, Hofmann A, Maaser A, Sumer S, Khudayberdiev S, Muhleisen TW (2015). Genome-wide analysis implicates microRNAs and their target genes in the development of bipolar disorder. Transl Psychiatry.

[33] Banach E, Dmitrzak-Weglarz M, Pawlak J, Kapelski P, Szczepankiewicz A, Rajewska-Rager A (2017). Dysregulation of miR-499, miR-708 and miR-1908 during a depression episode in bipolar disorders. Neurosci Lett.

[34] Strazisar M, Cammaerts S, van der Ven K, Forero DA, Lenaerts AS, Nordin A (2015). MIR137 variants identified in psychiatric patients affect synaptogenesis and neuronal transmission gene sets. Mol Psychiatry.

[35] Schizophrenia Working Group of the Psychiatric Genomics Consortium. (2014). Biological insights from 108 schizophrenia-associated genetic loci. Nature.

[36] Anttila V, Bulik-Sullivan B, Finucane HK, Walters RK, Bras J, Duncan L (2018). Analysis of shared heritability in common disorders of the brain. Science.

[37] O’Leary NA, Wright MW, Brister JR, Ciufo S, Haddad D, McVeigh R (2016). Reference sequence (RefSeq) database at NCBI: current status, taxonomic expansion, and functional annotation. Nucleic Acids Res.

[38] de Leeuw CA, Mooij JM, Heskes T, Posthuma D (2015). MAGMA: generalized gene-set analysis of GWAS data. PLoS Comput Biol.

[39] McGeary SE, Lin KS, Shi CY, Pham TM, Bisaria N, Kelley GM, (2019). The biochemical basis of microRNA targeting efficacy. Science.

[40] Miller JA, Ding SL, Sunkin SM, Smith KA, Ng L, Szafer A (2014). Transcriptional landscape of the prenatal human brain. Nature.

[41] Treutlein J, Cichon S, Ridinger M, Wodarz N, Soyka M, Zill P (2009). Genome-wide association study of alcohol dependence. Arch Gen Psychiatry.

[42] Untergasser A, Cutcutache I, Koressaar T, Ye J, Faircloth BC, Remm M (2012). Primer3—new capabilities and interfaces. Nucleic Acids Res.

[43] Sherry ST, Ward MH, Kholodov M, Baker J, Phan L, Smigielski EM (2001). dbSNP: the NCBI database of genetic variation. Nucleic Acids Res.

[44] Lorenz R, Bernhart SH, Honer Zu Siederdissen C, Tafer H, Flamm C, Stadler PF (2011). ViennaRNA Package 2.0. Algorithms Mol Biol.

[45] Valluy J, Bicker S, Aksoy-Aksel A, Lackinger M, Sumer S, Fiore R (2015). A coding-independent function of an alternative Ube3a transcript during neuronal development. Nat Neurosci.

[46] Schratt GM, Nigh EA, Chen WG, Hu L, Greenberg ME (2004). BDNF regulates the translation of a select group of mRNAs by a mammalian target of rapamycin-phosphatidylinositol 3-kinase-dependent pathway during neuronal development. J Neurosci.

[47] Baker JM, Boyce FM. High-throughput functional screening using a homemade dual-glow luciferase assay. J Vis Exp. 2014;50282. 10.3791/50282. 10.3791/50282 PMC4186351 24962249

[48] Khudayberdiev SA, Zampa F, Rajman M, Schratt G (2013). A comprehensive characterization of the nuclear microRNA repertoire of post-mitotic neurons. Front Mol Neurosci.

[49] Liu JZ, McRae AF, Nyholt DR, Medland SE, Wray NR, Brown KM (2010). A versatile gene-based test for genome-wide association studies. Am J Hum Genet.

[50] Schizophrenia Psychiatric Genome-Wide Association Study (GWAS) Consortium. (2011). Genome-wide association study identifies five new schizophrenia loci. Nat Genet.

[51] Kwon SC, Baek SC, Choi YG, Yang J, Lee YS, Woo JS (2019). Molecular basis for the single-nucleotide precision of primary microRNA processing. Mol Cell.

[52] Nguyen TA, Jo MH, Choi YG, Park J, Kwon SC, Hohng S (2015). Functional anatomy of the human microprocessor. Cell.

[53] Martins HC, Gilardi C, Sungur AÖ, Winterer J, Pelzl MA, Bicker S, et al. Bipolar-associated miR-499-5p controls neuroplasticity by downregulating the Cav1.2 subunit CACNB2. EMBO Rep. (in the press). 10.15252/embr.202154420 PMC9535808 35969184

[54] Simms BA, Zamponi GW (2014). Neuronal voltage-gated calcium channels: structure, function, and dysfunction. Neuron.

[55] Heyes S, Pratt WS, Rees E, Dahimene S, Ferron L, Owen MJ (2015). Genetic disruption of voltage-gated calcium channels in psychiatric and neurological disorders. Prog Neurobiol.

[56] Harrison PJ, Hall N, Mould A, Al-Juffali N, Tunbridge EM (2021). Cellular calcium in bipolar disorder: systematic review and meta-analysis. Mol Psychiatry.

[57] Forstner AJ, Basmanav FB, Mattheisen M, Bohmer AC, Hollegaard MV, Janson E (2014). Investigation of the involvement of MIR185 and its target genes in the development of schizophrenia. J Psychiatry Neurosci.

[58] Guo L, Lu Z (2010). The fate of miRNA* strand through evolutionary analysis: implication for degradation as merely carrier strand or potential regulatory molecule?. PLoS ONE.

[59] McCreight JC, Schneider SE, Wilburn DB, Swanson WJ (2017). Evolution of microRNA in primates. PLoS ONE.

[60] Duan J, Shi J, Fiorentino A, Leites C, Chen X, Moy W (2014). A rare functional noncoding variant at the GWAS-implicated MIR137/MIR2682 locus might confer risk to schizophrenia and bipolar disorder. Am J Hum Genet.

[61] Fiorentino A, O’Brien NL, Sharp SI, Curtis D, Bass NJ, McQuillin A (2016). Genetic variation in the miR-708 gene and its binding targets in bipolar disorder. Bipolar Disord.

[62] Bansal V, Libiger O, Torkamani A, Schork NJ (2010). Statistical analysis strategies for association studies involving rare variants. Nat Rev Genet.

[63] O’Roak BJ, Vives L, Fu W, Egertson JD, Stanaway IB, Phelps IG (2012). Multiplex targeted sequencing identifies recurrently mutated genes in autism spectrum disorders. Science.

[64] Devanna P, Chen XS, Ho J, Gajewski D, Smith SD, Gialluisi A (2018). Next-gen sequencing identifies non-coding variation disrupting miRNA-binding sites in neurological disorders. Mol Psychiatry.

